# Editorial: Generating and Sustaining Stable Autoantigen-Specific CD4 and CD8 Regulatory T Cells in Lupus

**DOI:** 10.3389/fimmu.2022.838604

**Published:** 2022-03-07

**Authors:** Syamal K. Datta, David A. Horwitz, Ciriaco A. Piccirillo, Antonio La Cava

**Affiliations:** ^1^ Department of Medicine, Division of Rheumatology, Northwestern University Feinberg School of Medicine, Chicago, IL, United States; ^2^ General Nanotherapeutics, LLC, Santa Monica, CA, United States; ^3^ Department of Medicine, Keck School of Medicine, University of Southern California, Los Angeles, CA, United States; ^4^ Department of Microbiology and Immunology, McGill University, Montreal, QC, Canada; ^5^ Program in Infectious Diseases and Immunology in Global Health Research Institute, McGill University, Montreal, QC, Canada; ^6^ Department of Medicine, University of California Los Angeles, Los Angeles, CA, United States

**Keywords:** autoimmunity, systemic lupus erythematosus, T regulatory cells, immune tolerance, peptide epitopes, graft-versus-host disease, nanoparticle (NP), humanized mice

## CD4^+^ and CD8^+^ Regulatory T Cells in Lupus

Systemic lupus erythematosus (lupus) is a complex autoimmune disease that involves major components of the immune system. It has heterogeneous clinical manifestations and, at the earliest stages, it is characterized by a deficiency of IL-2 and TGF-β, (1, 2), and epigenetic abnormalities that include an abnormal development and stability of CD4^+^CD25^+^CD127^low^ T regulatory (Treg) cells (3). However, merely enumerating the levels of circulating Treg cells in lupus patients has yielded inconsistent results because some of those Treg cells are functionally inactive (4, 5), and Treg cells directed to major autoantigens of lupus are not detectable in patients with active disease (6) and Robinson et al. Therefore, generating stable Treg cells that preferentially suppress pathogenic activity of self-reactive immune cells represents a critical therapeutic goal for the modulation of lupus disease, as discussed in this Research Topic.

Datta reviews the origins of the first experiments that showed that an endogenous self-antigen, namely nucleosomes from apoptotic cells, linked self-reactive lupus T helper (Th) and B cell with cognate interactions leading to the production of class-switched nephritogenic anti-dsDNA autoantibodies. Subsequently, minute doses of certain histone peptide epitopes from nucleosomes were found to induce autoantigen-specific CD4^+^ and CD8^+^ Treg cells. Surprisingly the epitopes were also found to render dendritic cells tolerogenic directly, which led to inhibition of multiple autoreactive cells participating in pathogenic autoimmune response in lupus.

Wei et al. review cellular mechanisms that lead to production of high-affinity autoantibodies in SLE. The onset of autoantibodies in systemic autoimmunity requires a complex and highly regulated B-T cell functional crosstalk as well as mature germinal center (GC) formation in B cell follicles of secondary lymphoid tissues. A key regulator of such events is the T follicular regulatory cell (TFR), a specialized Treg cell population that protects from hyperactivity of self-reactive T and B cells. However, recent studies show that TFRs manifest functional plasticity as they can lose Foxp3 expression and convert into disease-promoting “ex-TFRs” that acquire potent effector/inflammatory functions. The authors review currently proposed intrinsic and extrinsic mechanisms of regulation of TFR function and discuss the roles of TFR plasticity in autoantibody production in the pathophysiology of SLE.

Singh et al. describe a Treg cell-inducing peptide called pCons that was derived from V-region sequences of anti-dsDNA autoantibodies. Singh et al. also describe the gene expression profiles and immunological markers of pCons-induced CD8^+^ Treg cells in NZB/W lupus mice, discussing potentially interesting functional features of those pCons-induced CD8^+^ Treg cells in the downregulation of lupus autoimmunity.

Kato and Perl critically elaborate on the recent exciting findings that showed a peripheral expansion of Treg cells in lupus patients treated with low-dose IL-2. The authors report that although IL-2 does induce CD4^+^ Treg cells in lupus patients, in the meantime this cytokine also promotes an expansion of CD8^+^ T cells that produce pro-inflammatory IFN-γ. This new finding raises questions on how to optimize treatments with IL-2 in lupus patients to avoid unwanted side effects while promoting the Treg cell-mediated beneficial activities on disease manifestations.

Papillion and Ballesteros-Tato point out that although IL-2 normally inhibits T follicular helper cells in SLE, IL-6 blocks this inhibitory effect, documenting that IL-6 blocked the upregulation of IL-2Rβ (CD122).

Hatzioannou et al. review the two-faced role and plasticity of Treg cells in autoimmunity and cancer, focusing on the phenotypic characteristics that Treg cells display in association with their functional flexibility. The authors elaborate on the potential therapeutic implications of targeting Treg cell plasticity either to maintain functional stability in Treg cell control of immune effector responses that sustain chronic inflammation, or to favor Treg cell destabilization in the fight against cancer.

Horwitz et al. and Robinson and Thomas review an emerging approach that avoids the use of corticosteroids and immunosuppressive drugs to treat SLE. Nanoparticles (NPs) engineered to target specific cell populations are used to repair defects in the homeostatic immune regulation, and to restore immune tolerance to autoantigens. NPs can be targeted to antigen presenting cells (APCs) to switch them from supporting pathogenic T cells to favoring the expansion of therapeutic Treg cells. Alternatively, NPs can be targeted directly to T cells for the induction and expansion of CD4^+^ and CD8^+^ Treg cells. Both reviews discuss potential approaches to induce autoantigen specific Treg cells, without antigen or with peptide antigens. Attention is given to lupus-specific peptide autoantigens to induce antigen-specific Treg cells and the use of NPs to function as artificial APCs (aAPCs) that induce multiple populations of lymphocytes to become regulatory cells.

Giang et al. demonstrate that aAPCs made of NPs that provide IL-2 and TGF-β to human cells can induce human CD4^+^ and CD8^+^ FoxP3^+^ Treg cells both *in vitro* and *in vivo* in immunodeficient mice. These human Treg cells protect the mice from human anti-mouse graft versus host disease (GvHD). These studies suggest the possibility that repairing both the IL-2 and TGF-β defects may be necessary for a sustained disease remission in SLE.

## Conclusions

This Research Topic provides updates on the influences exerted by CD4^+^ and CD8^+^ Treg cells on the pathogenesis and modulation of SLE disease manifestations. Among the possibilities of Treg cell modulation, epigenetic manipulations that could improve Treg cell stability and function are actively investigated and expected to be tested in clinical trials (7, 8). Several immunotherapeutic studies that focused on the numeric and functional modulation of CD4^+^ and CD8^+^ Treg cells, and have yielded promising laboratory results, are moving to the patient’s bedside. The restoration and maintenance of Treg cell predominance over effector cells has the potential to promote long-term remission of autoimmune disease, and ultimately prevent autoimmunity in susceptible individuals.

## Author Contributions

All authors wrote and revised this Editorial and have approved this version for submission. SD initially conceived of, and established this Research Topic.

## Conflict of Interest

DAH is the CEO of and holds stock in General Nanotherapeutics, LLC.

The remaining authors declare that the research was conducted in the absence of any commercial or financial relationships that could be construed as a potential conflict of interest.

## Publisher’s Note

All claims expressed in this article are solely those of the authors and do not necessarily represent those of their affiliated organizations, or those of the publisher, the editors and the reviewers. Any product that may be evaluated in this article, or claim that may be made by its manufacturer, is not guaranteed or endorsed by the publisher.
